# How Much Is Our Fairness Worth? The Effect of Raising Stakes on Offers by Proposers and Minimum Acceptable Offers in Dictator and Ultimatum Games

**DOI:** 10.1371/journal.pone.0060966

**Published:** 2013-04-08

**Authors:** Julie Novakova, Jaroslav Flegr

**Affiliations:** Biology Department, The Faculty of Science, Charles University, Prague, Czech Republic; Hungarian Academy of Sciences, Hungary

## Abstract

**Background:**

The aim of this study was to determine whether people respond differently to low and high stakes in Dictator and Ultimatum Games. We assumed that if we raised the stakes high enough, we would observe more self-orientated behavior because fairness would become too costly, in spite of a possible risk of a higher punishment.

**Methods:**

A questionnaire was completed by a sample of 524 university students of biology. A mixed linear model was used to test the relation between the amount at stake (CZK 20, 200, 2,000, 20,000 and 200,000, i.e., approximately $1–$10,000) and the shares, as well as the subjects’ gender and the design of the study (single vs. multiple games for different amounts).

**Results:**

We have discovered a significant relationship between the amount at stake and the minimum acceptable offer in the Ultimatum Game and the proposed shares in both Ultimatum and Dictator Games (p = 0.001, p<0.001, p = 0.0034). The difference between playing a single game or more games with several amounts at stake did not influence the relation between the stakes and the offered and minimum acceptable shares. Women proved significantly more generous than men in their offers in the Dictator Game (p = 0.007).

**Conclusion:**

Our results suggest that people’s behavior in the Dictator and Ultimatum Games depends on the amount at stake. The players tended to lower their relative proposed shares, as well as their relative minimum acceptable offers. We propose that the Responders’ sense of equity and fair play depends on the stakes because of the costs of maintaining fairness. However, our results also suggest that the price of fairness is very high and that it is very difficult, probably even impossible, to buy the transition of *Homo sociologicus* into *Homo economicus.*

## Introduction

The Ultimatum Game was designed by Güth, Schmittberger and Schwarze [1] to study strategies in bargaining under a specific kind of ultimatum. The game involves two experimental subjects, usually called the Proposer and the Responder. The Proposer is given a certain amount by the experimenter which he can divide between themself and the Responder. The Responder can either accept their share or reject it. In case of rejection, neither of them gets anything from the initial amount at stake. In case of acceptance, each gets the share offered by the Proposer. In most experiments, the subjects do not know or see each other and all contact between them is mediated by the experimenter. Also, under normal circumstances, the game is played without repetition.

A logical solution for the Proposer in the Ultimatum Game would be proposing the smallest possible positive share; for the Responder, it would be logical to accept any non-zero share. However, in real experiments, people do not behave as profit-maximizers and the Responders often reject a non-zero offer if it seems unfair to them. The Proposers rarely offer the smallest amount possible, being able to imagine the rejection.

Mean offers are usually between 40 and 50% of the amount at stake, the modal offer being in most studies 50%. Offers of 20% or less are rejected more than half the time, those of 30% quite often too [2].

The Dictator Game [3], [4] is based on similar principles with one important difference: The Responder (in this case called the Recipient) cannot influence the outcome of the game and the Proposer (the Allocator) gets the share proposed by themself.

The Dictator Game (DG) creates an even greater paradox than the Ultimatum Game (UG): the Proposer has no reason to give away any part of the money; however, a vast majority of the Proposers give the Responders something, usually around 20% of the amount at stake [2], [4].

Multiple hypotheses have been proposed trying to explain the unexpected behavior in both the UG and the DG. Among the first was Matthew Rabin’s explanation [5], which introduced the concept of fairness into the games.

Levine [6] developed a model counting with several types of people on a scale from spiteful to altruistic, thus explaining the mostly fair-seeming but sometimes selfish, spiteful or illogical behavior.

Fehr and Schmidt [7] and Bolton and Ockenfels [8] came up with inequity aversion models; however, unlike Rabin’s, their hypothesis did not include the players’ intentions, only the comparison of the payoffs. This almost definitely amounts for a certain part of the decisions in the games, but as Güth and van Damme [9] showed by introducing a third “dummy” player into the UG, similar to the Recipient in the DG, the Responders indeed cared about equity – toward themselves. The relative payoffs of the “dummies” had no significant effect on the rejection rates.

So far, it seems that the players care about their co-players’ intentions, fairness to themselves and both the absolute and relative payoffs. Camerer and Thaler [10] commended Rabin’s attempts to include fairness and manners in game theory. And according to Falk, Fehr and Fischbacher [11], intentions of the co-player are the most important for most players, although they care about maximizing their payoff as well.

However, it looks like the Proposers want to *seem* fair rather than *be* fair. By manipulating the value of the chips used in their experiment instead of bills, Kagel, Kim and Moser [12] showed that many Proposers only offered what the Responders would think was the fair share, but actually was not. Although fairness is a very important social norm, it seems that for many people it is a norm only, but not something intrinsically incorporated in their behavior; if there is a chance of violating it without the other party knowing, many people do it.

Rotemberg [13] proposed an explanation of risk-loving for rejections of low offers and also proposing them; these strategies violate payoff maximization in most situations.

Finally, Levitt and List [14] argued that giving in the DG is a framing effect based on the fact that the situation evokes a social norm of giving and the subjects do not violate it, partly because of the authoritative figure of the experimenter. Nevertheless, that does not explain the findings from the UG very well.

In the 1990s, an important question was raised in Ultimatum and Dictator Games research: Would raising the stakes influence the players’ behavior – and if yes, then how?

The first notions counted on the assumption that the Responders would be unwilling to turn down an unfair offer, if it represented a lot of money, and the Proposers would realize it and lower their relative offers. Rejecting a substantial sum of money would be a very high price for satisfying one’s taste for fairness.

Game theorists, economists and evolutionary psychologists have since attempted to determine whether the players’ strategies change under high stakes. Most of them discovered no difference compared to low stakes; however, other studies found that with the stakes raised, the relative offers and their rejection rates go down, as shortly summarized below.

Tompkinson and Bethwaite [15] used a sample of 43 lawyers who filled out their questionnaire to determine whether there was a difference in the proposals and minimum acceptable offers (MAOs) under the stakes of $10 and $10,000 respectively. The mean offers did lower, but the modal offers were still 50% and the difference was not statistically significant.

Hoffman, McCabe and Smith [16] could not reject the null hypothesis of the players’ responses on the stakes of $10 and $100 respectively having the same mean.

Slonim and Roth [17] conducted an experiment in the Slovak Republic with the stakes of 60, 300 and 1 500 Slovak Crowns (about $2, $10 and $50 in 1994 when the study was conducted). They too could not find any significant difference.

Lisa Cameron [18] used the opportunity to raise the stakes by a factor of 40 by conducting the experiment in Indonesia. It did not change the offers.

Carpenter, Verhoogen and Burks [19] replicated the experiment performed by Hoffman et al. [16] with raising the stakes from $10 to $100 and also found no significant change.

However, Munier and Zaharia [20] observed that raising the stakes by a factor of 50 had an influence on the minimum acceptable offers, which lowered, although it did not alter the proposed offers significantly.

Recently, a study introducing high stakes in India was published [21]. The authors used stakes raised by a factor of 1 000 in case of the highest amount at stake, which was 20 000 Indian rupees (approximately $410). The highest stakes amounted to a little over an average year’s income in the area. The Proposers also received a message informing them about the rational decision being the lowest possible positive offer. Almost all of the offers under all stakes were under 30%. The proportion offered decreased significantly as the stakes increased and so did the rejection rates. This example shows us that raising the stakes very high, possibly with help of the Proposer’s framing, can lead to far more “selfish” behavior in the game.

If we look at other experimental economic games, we find evidence of the influence of high stakes in the Trust Game [22], where the senders sent relatively less if the stakes were high; however, the difference was not significant as regards the proportions sent back by the receivers, which corresponds with findings from the DG (see below). Kocher, Martinsson and Visser [23] found no significant difference in the case of the Public Goods Game.

In the DG, Forsythe et al. [4] studied whether the Allocators give the Recipients a different share from the stakes of $5 or $10 respectively; they did not. List and Cherry [24] used stakes of 20 and 100 dollars and found no significant change either. Nevertheless, the number of papers on this topic is much lower than in the case of the UG.

An interesting DG experiment was conducted by Blake and Rand [25], who supplied the role of large monetary stakes by highly valued stickers in a study focused on children. They found that 6-year-old children donated significantly more low-value than high-value stickers. This not only tells us something about fairness and equity preferences among young children, but also shows that in some cases, offers can be significantly more selfish under the high stakes condition.

In his meta-study of the DG, Engel [26] found the effect of raising stakes significant if he focused on the studies which manipulated the amounts at stake. The higher the stakes, the more the Allocators kept to themselves. The effect explained about 3.6% of the data variability.

For further and broader information, Camerer and Hogarth [27] reviewed the literature available on the subject of high monetary incentives in experimental economics. They concluded that high stakes considerably influence judgement- and decision-making tasks, but have little effect on bargaining games. According to them, raised incentives might not change the subjects’ understanding of the task or their self-interest.

The most frequent explanation of those results is risk aversion in the case of high incentives [28], [29]; however, that cannot account for the findings from the DG; therefore a taste for fairness, as introduced by Rabin [5], is likely to partially contribute.

According to our hypothesis, the offers and MAOs would lower if the amount at stake was high enough; metaphorically speaking, the *Homo sociologicus* could be turned into *Homo economicus* by raising the stakes. Only two of the aforementioned studies have dealt with amounts higher than 100 dollars. In the present study we raised the stakes up to CZK 200,000 (approximately $10,000).

It is true that on the other hand, the Proposers could be more careful in their offers because the possible punishment for an unfair offer would be higher. However, we assumed that not many Responders would be willing to pay so much themselves to punish an unfair, but still extremely valuable offer in case of raised incentives, and that the Proposers might be counting on that, being able to imagine their own decisions in the same situation, and offer smaller proportions of the whole amount than in case of low incentives – although they might not drop very rapidly because of the other factor at play, the risk aversion.

And although some of the Responders might find the same proportional offers smaller than 50% “more unfair” under the high stakes condition, which could create a bigger discomfort, they also might be pleased just by the actual amount to gain. That would still be for example CZK 20,000 (almost a month wage in the Czech Republic), if the Proposer split the amount 9∶1 in case of the highest stakes.

We also decided to study the effect of playing a single game versus five different games, each one with increasing amounts at stake. We assumed that seeing the raising stakes could push the subjects even more toward a more selfish behavior, because – in spite of *not* repeating the same game and having no feedback from a co-player – it could evoke a learning situation, similar to playing with repetitions, such as in Roth and Erev [30] who observed a lowering in offers when the UG was played repeatedly. While in a one-shot game, the players both consciously and unconsciously use principles from everyday life, which are useful in reality but not in the game, in the subsequent rounds of a repeated game they gradually learn strategies closer to the basic game-theoretical model of *Homo economicus*, better suited for an anonymous game. Playing several different games with raising amounts could have an analogous effect.

As far as we know, this is the first study to explore the players’ behavior with raising stakes four times (by a factor of 10, 100, 1,000 and 10,000) and under the following conditions: playing one game and playing several games with a different amount each time.

## Methods

### Conducting the Study

We used a questionnaire method because it enabled testing a large sample of subjects and offer very high stakes. The sample consisted of 524 undergraduate students of the Faculty of Science of the Charles University, their mean age being 20.88 (median 20, s.d. 2.11). 388 (71%) of the subjects were females. The students were invited to take part in the study during regular evolutionary biology courses. They signed the informed consent form and filled out the questionnaire immediately after a written test which was an optional part of the evolutionary biology exam. For previous usage of this sample recruitment method, see Flegr and Příplatová [31]. Approximately 90% of students of Evolutionary Biology and Microevolution & Macroevolution took part in the written exam and about 95% of them consented to take part in the study. The study was approved by the IRB Charles University, the Faculty of Science.

Rules of the Ultimatum and Dictator Games were explained to the subjects in the questionnaire as stated below:

“The Dictator Game is played non-repeatedly and anonymously by two players, the Allocator and the Recipient. The Allocator is given an amount which they can divide between themself and the Recipient (the smallest, indivisible unit being CZK 1). They both keep the proposed shares.”

“The Ultimatum Game is played non-repeatedly and anonymously by two players, the Proposer and the Responder. The Proposer is given an amount which they can divide between themself and the Responder (the smallest, indivisible unit being CZK 1). This game is different in the aspect that the Responder can influence it: If they accept the offer, both players get the proposed shares. If they reject it, both get nothing.”

The experimental questions were “How much would you offer in the Dictator Game?”, “How much would you offer in the Ultimatum Game?” and “What is the minimum offer you would accept in the Ultimatum Game?”.

Besides the effect of raising the stakes, we also tested two different treatments on our sample; in Treatment Five, 101 students were given a questionnaire in which they answered the questions for five different amounts: CZK 20, 200, 2,000, 20,000 and 200,000 ($1 = approximately CZK 20). Another 423 students in Treatment One were divided into five groups and each one provided answers in a game with just one of these amounts (which is why we chose a larger sample for the second treatment).

### Statistics

We used a mixed linear model in SPSS 18.00 (IBM corporation) to estimate the relationship between the amount at stake and the shares allocated, and also for assessing the influence of the two treatments and gender of the subjects. Within-subjects effects of multiple observations for one person in Treatment Five were filtered in the model. Results with p<0.05 in a two-sided test were considered significant. The analyzed dataset is available for download as a supplement of this paper, Dataset S1.

## Results

The mean relative offers and minimum acceptable shares gradually lowered with the increasing stakes, as listed in Table 1.

**Table 1:** Mean offers in the DG and the UG.

	Mean offer in the DG	Mean offer in the UG	Mean MAO in the UG
CZK 20	28.3%	45.8%	41.9%
CZK 200	27.9%	43.4%	37.9%
CZK 2,000	24.7%	40.0%	34.7%
CZK 20,000	23.6%	38.7%	33.4%
CZK 200,000	23.3%	37.2%	30.1%

Mean offers in the Dictator and Ultimatum Games and minimum acceptable offers in the Ultimatum Game (expressed as a percentage of the amount at stake).

**Figure 1:**
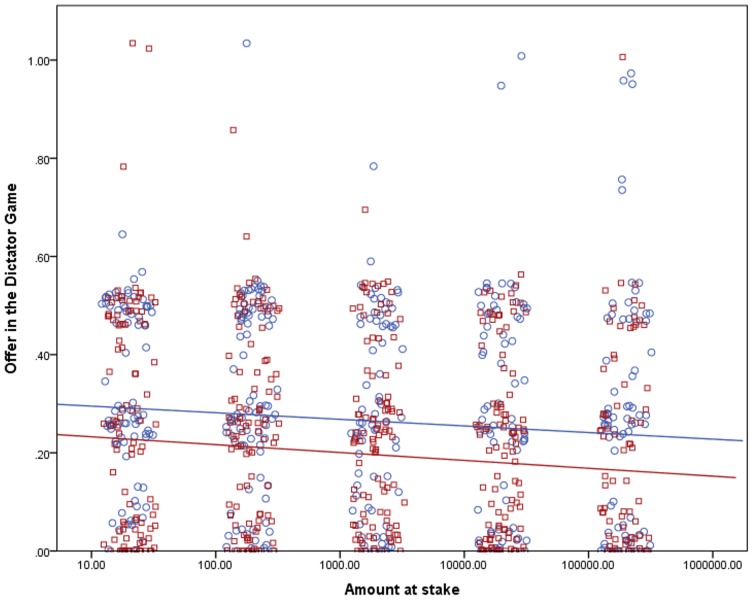
Effect of the amount at stake on the relative proposed shares in the DG. The stakes are shown on a logarithmic scale and are in CZK. The points were jittered to be better visible in their numbers. Treatment One, where the subjects only answered questions for one amount at stake, is marked with circles and the color of the points and the regression line is blue. The estimated effect size for the influence of the stakes on the shares in Treatment One is 0.8%. Treatment Five, where the subjects answered questions for all five amounts, is marked with squares and it has red points and the regression line. The estimated effect size for the influence of the stakes on the shares in Treatment Five is 1.2%.

**Figure 2:**
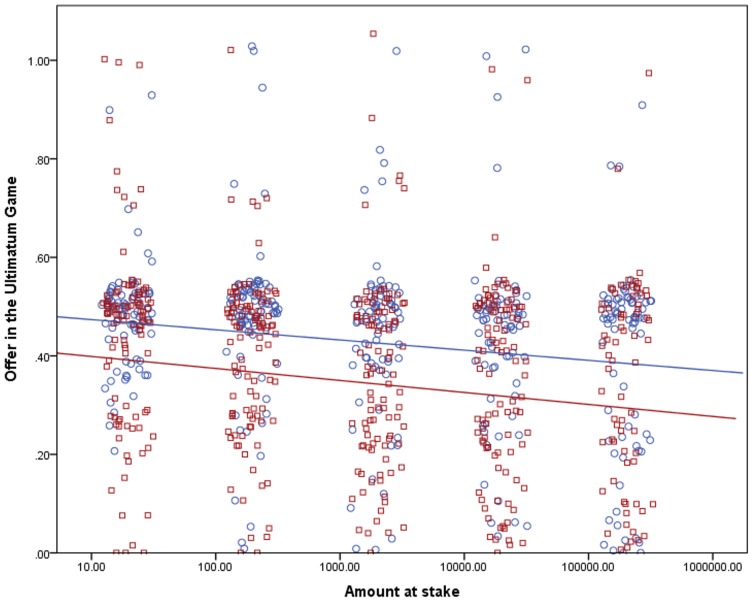
Effect of the amount at stake on the relative proposed shares in the UG. The stakes are shown on a logarithmic scale and are in CZK. The points were jittered to be better visible in their numbers. Treatment One, where the subjects only answered questions for one amount at stake, is marked with circles and the color of the points and the regression line is blue. The estimated effect size for the influence of the stakes on the shares in Treatment One is 3.2%. Treatment Five, where the subjects answered questions for all five amounts, is marked with squares and it has red points and the regression line. The estimated effect size for the influence of the stakes on the shares in Treatment Five is 3.0%.

**Figure 3:**
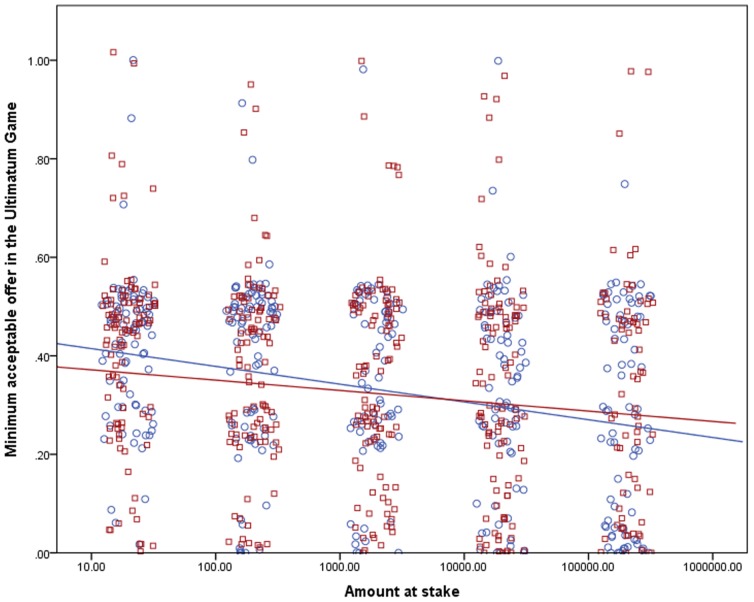
Effect of the amount at stake on the relative MAOs in the DG. The stakes are shown on a logarithmic scale and are in CZK. The points were jittered to be better visible in their numbers. Treatment One, where the subjects only answered questions for one amount at stake, is marked with circles and the color of the points and the regression line is blue. The estimated effect size for the influence of the stakes on the shares in Treatment One is 7.5%. Treatment Five, where the subjects answered questions for all five amounts, is marked with squares and it has red points and the regression line. The estimated effect size for the influence of the stakes on the shares in Treatment Five is 1.8%.

The dependent variables in the LMM analysis were the relative shares offered in the DG and the UG and the relative MAOs in the UG; the explanatory variables were a logarithm of the amount at stake and two binary variables, the treatment (one amount vs. five amounts at stake) and gender. The logarithmic transformation was used because the average shares (as seen in Table 1) dropped approximately the same for each step, not still more as the stakes rose; therefore we deemed this transformation useful to describe the influence of the stakes on the shares correctly. We included the subjects’ gender in the model because men and women had proven to behave differently in experimental economics games (as discussed in the next section) – and hence the gender could explain a part of the variability of the data. We estimated the influence of the treatment on the effect of the amount at stake on the offers and MAOs by including an interaction between the treatment and the amount at stake in the model. The mixed linear model provides an estimation of parameters of the line best fitting to describe the relationships between tested variables; however, it cannot provide the effect size. Therefore we also calculated an estimated effect size from a linear regression analysis for each treatment (see Figures 1, 2, and 3).

We found a significant negative relationship between the logarithm of the amount at stake and offers in the DG (*p* = 0.034), proposed shares in the UG (*p*<0.001) and MAOs in the UG (*p* = 0.001) as listed in Table 2 and shown in Figures 1 to 3. The effect of the treatment was insignificant, as well as the interactions of the treatment and the stakes. In the DG, women proved significantly more generous in their offers than men (*p* = 0.007); this effect was not observed in the UG. An analysis without interactions was run too to estimate how the treatment influenced the shares directly – not how it influenced the relation between them and the stakes. In this case, the effect of the treatment was significant in offers in both games (*p*<0.001 in both cases); the effects of the stakes and in the DG also gender remained. In MAOs in the UG, the effect of the treatment on the shares was insignificant (*p* = 0.567).

**Table 2:** Influence of the stakes on the offers.

		Estimate	Standard error	df	t	p value
Offer in theDictator Game	*Intercept*	*0.253*	*0.027*	*555.238*	*9.261*	*<0.001*
	*Logarithm of the stakes*	*− 0.016*	*0.007*	*541.308*	*− 2.128*	*0.034*
	Treatment	0.045	0.036	459.864	1.261	0.208
	Treatment * Logarithm of the stakes	0.002	0.011	523.646	0.158	0.874
	*Gender*	*0.044*	*0.022*	*814.412*	*2.724*	*0.007*
Offer in theUltimatum Game	*Intercept*	*0.453*	*0.022*	*506.526*	*20.583*	*<0.001*
	*Logarithm of the stakes*	*− 0.027*	*0.006*	*517.635*	*− 4.566*	*<0.001*
	Treatment	0.045	0.028	410.276	1.583	0.114
	Treatment * Logarithm of the stakes	0.007	0.009	497.794	0.794	0.428
	Gender	0.023	0.014	813.467	1.707	0.088
Minimum acceptable offerin the Ultimatum Game	*Intercept*	*0.434*	*0.024*	*485.479*	*17.957*	*<0.001*
	*Logarithm of the stakes*	*− 0.023*	*0.007*	*508.084*	*− 3.406*	*0.001*
	Treatment	0.031	0.031	390.537	1.011	0.313
	Treatment * Logarithm of the stakes	− 0.013	0.010	486.923	− 1.391	0.165
	Gender	0.008	0.015	792.031	0.544	0.587

This table conveys estimates of the relationship between the shares and the stakes plus treatments and gender and the interactions of treatment with other explanatory variables. Significant results (p<0.05) are shown in italics.

## Discussion

We found that the amount at stake had a significant effect on the subjects’ behavior in the games; the more was at stake, the relatively lower were the proposals as well as the minimum acceptable offers. This supports our hypothesis that high stakes can lower people’s equity preference and their tendency to show fair play; the price to be paid for fairness would be too high and most of our subjects seemed unwilling to pay it, as they were willing to accept much lower relative offers with the amount at stake growing. Especially the MAOs lowered quite quickly and the subjects seemed to be quite readily accepting unfair offers (albeit not very often less than 20%).

This is in contrast with the results of a number of previous studies [15]–[19], although partially in correspondence with some others (Munier and Zaharia [20] in the case of MAOs in the UG; Blake and Rand [25] in the case of the given share in the DG, which proved influenced by the value of the stickers in a part of their sample) and fully in line with Andersen et al. [21].

Our proposed explanation is that with raising the stakes high enough, specifically four times by the factor of ten, the subjects would be unwilling to pay the price for fairness which would be up to CZK 100,000 in the case of the largest stakes. Many studies showing an absence of the effect used a smaller range of stakes which resulted into a too low effect size.

Of course if the interactions between the players were repeated, then punishing the Proposer for an unfair offer by turning it down would be a functional profit-maximizing strategy from the Responder, which would most probably receive a better offer next time. This would lead to a “fairness spread” in a population, which is with a high probability a process actually running in human societies. Also the results from the Spatial Ultimatum Game [32] suggest that. Nevertheless, we focused on a “classic” Ultimatum Game, which tries to eliminate the social conventions by a one-round anonymous setting.

Another reason why we might have discovered the effect is a large sample; most studies in the field worked with a much smaller sample, while we had 524 participants. Such a sample enabled a higher statistical power of the tests and could have contributed to the rejection of the null hypothesis.

Most important is to compare our results with the opposite results reported by Tompkinson and Bethwaite [15] who used the amount of $10,000 in their study. We suggest three most probable explanations: Whereas their sample consisted of established lawyers, ours were university students of biology. Although some studies [33] found no significant difference between students and other demographic samples in experimental economics games, the fact that students usually have very low incomes might have contributed to the fact that they were willing to accept lower relative offers, could imagine other colleagues doing so and therefore offered less to maximize their own profit. This assumption is supported by the findings of Engel [26] about students being less generous than working people (see more in Limitations).

Another reason might be the fact that the average wage in the Czech Republic is about $14,500 p.a. In the USA, it was around $35,000 in 1993, and approximately $62,500 in 2010, according to the U.S. Consumer Expenditure Interview Survey. If an experiment with $10,000 was conducted in the USA today, it would still mean four times less for the US citizens than for the Czech people – as if the experiment was conducted with $2,500 instead of $10,000. Tompkinson’s and Bethwaite’s [15] stakes of $10,000 are less than a third of the US average wage in 1993. Our stakes of $10,000 correspond to two thirds of an average wage in the Czech Republic, so it cannot be very well compared. The stakes would have to have been $20,000 in the US in 1993 to have a similar value for the experimental subjects. That is a very large difference and as far as we know, no one has carried out a study with $20,000 at stake in the United States.

The third reason concerns the sample size; as mentioned above, our sample of 524 participants is much larger than usual. Tompkinson and Bethwaite had a sample consisting of 43 people. Also, the intensity of the effect in our study is not big, the shares did not drop rapidly with raising the stakes and the percentage of explained variability is not high. In a small sample, such an effect might not be detected as significant despite its presence.

The absence of a significant effect of the treatment on the relationship between the stakes and the relative shares rejected our hypothesis that in Treatment Five, the subjects would lower their shares more rapidly. The treatment alone had an effect on offers made by the Allocators and Proposers, which were lower in Treatment Five, but the slope of the line fit to the shares decreasing with the increasing amount at stake remained practically the same in both treatments.

In differences between both genders, women proved more generous in the Dictator Game; as giving in the Dictator Game is more of an expression of altruism, while the Ultimatum Game is more like bargaining, it is possible that this fact might have contributed to the differences in the Dictator Game and not in the Ultimatum Game.

### Limitations

It could be supposed that in a questionnaire study, the subjects had no reason to be honest about their real behavior; however, Güth et al. [1] found no significant difference between results from experiments with hypothetical and real stakes in their initial UG experiment. Camerer and Hogarth [27] mentioned in their review of the use of high incentives in experimental games that the Allocators usually kept more for themselves when the monies were real. Cameron [18] found the Proposers greedier in hypothetical games under high stakes than in real-money games; the Responders seemed more fairness-oriented in games with hypothetical money, more willing to turn down unfair offers.

Hertwig and Ortmann [34] investigated the effect of financial incentives in psychological and economic studies published in the *Journal of Behavioral Decision Making*. The results were inconsistent; in various cases, the incentives reduced data variability, decreased an effect of framing, influenced people’s judgement, brought decisions closer to a modelled situation, impaired subjects’ performance or had no effect at all. However, they focused mainly on tasks of judgement and decision.

Lindová et al. [35] conducted a real-money Dictator Game experiment with a similar sample (biology students at the Faculty of Science of Charles University in Prague) as we had with our hypothetical stakes and the Allocators gave the Recipients CZK 3.19 on average from the whole amount of CZK 10. The share of 31.9% is quite consistent with our results of 28.3% for CZK 20. The proportion given from CZK 10 is only a little higher, which supports the hypothesis that with rising stakes, the shares lower accordingly.

Engel [26] performed a meta-analytical study of 131 papers on the DG. He found the effect of asking hypothetical questions indistinguishable from real incentives at stake. Amir et al. [36] found that stakes of $1, compared to zero stakes, significantly reduced the mean offer in the DG, but in contrast, caused a marginally significant increase in the UG. Given the significance of 9.7% and a small effect size, the authors concluded that stakes have only little effect on the proposals in the UG. There was no significant effect found in the case of the MAOs in the UG. On the basis of these studies, we can assume that the hypothetical stakes have most likely a minor or negligible effect on the subjects’ answers. Using hypothetical instead of real stakes also had two large advantages – it enabled us to use very large amounts at stake and helped us achieve an absolutely blind design of the study.

In our study, we worked with student participants; it could be supposed that such a homogeneous sample tells little about the behavior of the majority of people. It was discovered, for example, that more educated people tend to be more generous [37], [38]. However, regardless of the initial proposals and acceptable offers, according to our hypothesis the relative shares would lower with raising the amounts at stake, therefore having students as our sample represented no complication in determining the effect of stakes correctly. Moreover, what we actually found was an opposite of what might be expected based on this allegation; our subjects appeared less generous than people in most studies and their generosity lowered as the stakes grew. Bekkers [33] found in his broad study no difference in giving in the DG between students and non-students; it seems that the effect of education comes after graduation, most probably because the graduates’ income increases once they enter the workforce.

In his large meta-analysis, Engel [26] found that students usually give less and are more likely to give nothing in the DG. This contradicts the initial assumptions that students, who have little experience with a harsh economic reality, would behave more altruistically than people from the professional environment. However, it is more fit to describe our results. It is also possible that the fact that the students filled out their questionnaires after an exam contributed to their more selfish behavior, because they might have been tired or stressed.

The majority of our subjects were female, who tended to be more generous, similarly as in a study conducted by Eckel and Grossman [39] or in the meta-analysis by Engel [26] in an analysis of studies reporting subjects’ gender in the DG; nevertheless, the same as stated above applies here – the possible higher generosity of the sample represented no complication in studying whether there would be a trend of lowering the offers and MAOs while raising the stakes. The connection between gender and generosity proved significant in our study for the offers in both games.

It is not clear to what extent the results obtained from a Czech population could be applied to people in general. The Czech Republic is a post-communist country, at the time of our research twenty-two years after the transition to democracy. It is also one of the most atheistic countries in the world. It would be important to repeat our study in other countries in the future. We should not forget that cultural diversity could lead to very different results in some cases. Behavior of players in WEIRD (Western, Educated, Industrialized, Rich and Democratic) countries can differ substantially from players in developing countries or members of completely non-industrial societies. Henrich et al. [40] showed us in his comparison that the United States of America are on the most generous end of the spectre and there can be a more than 20% gap between the DG and UG offers in different societies.

### Conclusion

Unlike the majority of previous studies, we found a significant difference between the proposals and minimum acceptable offers in the Dictator and Ultimatum Games under different amounts at stake. According to our hypothesis, raising the stakes high enough should give the players enough reason to deviate from the norms of fairness, and both offer and accept lower relative offers. However, the results are still very far from the “truly selfish” subgame perfect equilibrium.

Besides raising the stakes by a factor of 10 four times (therefore by a factor of 10,000 in case of the highest stakes), the most probable reasons that might have contributed to this finding include the questionnaire method (which was, however, necessary in this case and other studies found it indistinguishable from using real monetary stakes) and a large sample which could have allowed to detect effects that would not prove significant in too small a sample.

Our study showed that the shares in the Ultimatum and Dictator Games decreased with the increasing amount of money in stake. This suggests that the taste for fairness can be at least partly traded for money. However, our results also showed that the shares did not drop too much, so that the price of fairness is relatively high and it is very difficult, probably even impossible, to fully buy the transition of *Homo sociologicus* into *Homo economicus*. It would of course be very desirable to support this optimistic conclusion by obtaining the same results in a further real-money study.

## Supporting Information

Dataset S1 These data were used in the analysis described in the current paper. It contains the following variables: identification, dictator_offer, ult_offer, ult_accept, amount, Treatment, logamount and sex_num. Identification indicates the subject from our sample of 524 people, which is necessary in Treatment Five, where the same subject played five games with different incentives. Dictator_offer is a share offered by the subject in the Dictator Game, ult_offer shows a share offered in the Ultimatum Game, ult_accept means the minimum acceptable share in the Ultimatum Game. Amount indicates the amount at stake in every particular game. Treatment denotes the treatment used in the game – One or Five. Logamount is the logarithm of the amount at stake. Sex_num indicates the gender of the subject, expressed as a number, where 0 means female and 1 male.
(SAV)
